# The Effect of Surrounding Vegetation on Basal Stem Measurements Acquired Using Low-Cost Depth Sensors in Urban and Native Forest Environments

**DOI:** 10.3390/s23083933

**Published:** 2023-04-12

**Authors:** James McGlade, Luke Wallace, Bryan Hally, Karin Reinke, Simon Jones

**Affiliations:** 1 School of Science, Royal Melbourne Institute of Technology Univeristy, 124 La Trobe St, Melbourne, VIC 3000, Australia; 2 School of Geography, Planning and Spatial Sciences, University of Tasmania, Churchill Ave, Hobart, TAS 7001, Australia

**Keywords:** RGB-D, forestry, inventory, low-cost, remote sensing

## Abstract

Three colour and depth (RGB-D) devices were compared, to assess the effect of depth image misalignment, resulting from simultaneous localisation and mapping (SLAM) error, due to forest structure complexity. Urban parkland (S1) was used to assess stem density, and understory vegetation (≤1.3 m) was assessed in native woodland (S2). Individual stem and continuous capture approaches were used, with stem diameter at breast height (DBH) estimated. Misalignment was present within point clouds; however, no significant differences in DBH were observed for stems captured at S1 with either approach (Kinect *p* = 0.16; iPad *p* = 0.27; Zed *p* = 0.79). Using continuous capture, the iPad was the only RGB-D device to maintain SLAM in all S2 plots. There was significant correlation between DBH error and surrounding understory vegetation with the Kinect device (*p* = 0.04). Conversely, there was no significant relationship between DBH error and understory vegetation for the iPad (*p* = 0.55) and Zed (*p* = 0.86). The iPad had the lowest DBH root-mean-square error (RMSE) across both individual stem (RMSE = 2.16 cm) and continuous (RMSE = 3.23 cm) capture approaches. The results suggest that the assessed RGB-D devices are more capable of operation within complex forest environments than previous generations.

## 1. Introduction

Proximal three-dimensional (3D) remote sensing approaches, have revolutionised the acquisition of forest inventories, allowing for rapid and accurate measures of biophysical traits, to support the sustainable management of forests [1,2]. The adoption of this technology can largely be attributed to continued improvements in light detection and ranging (LiDAR) technologies since the early 2000s [3,4]. Proximal 3D remote sensing in forestry, involves close-range (≤100 m) measurements of environmental features, from both terrestrial and airborne perspectives. Recently, proximal LiDAR approaches have begun to see operational adoption, replacing or supplementing visual and conventional tool assisted methods (e.g., calipers), that are often time consuming and/or lacking in quantitative measurement accuracy [5,6,7,8]. Furthermore, the detailed representations of biophysical structures they provide, present the opportunity for the development of new metrics to describe forests [3,9].

The ground-based 3D remote sensing assessment of forest attributes—such as stem location, basal diameter and vertical forest structure—was initially facilitated through the employment of terrestrial laser scanning (TLS), which now forms the standard that alternative ground-based 3D remote sensing technologies are currently compared to [4]. The continued development of LiDAR hardware and computational software, has led to the exploration of personal laser scanning (PLS) and drone-mounted LiDAR approaches for forest measurement tasks, that aim to overcome the limitations of long capture times, occlusion, and registration error associated with the static nature of TLS [10,11,12,13].

Regardless, TLS and alternative conventional LiDAR technologies have a high associated hardware cost that, whilst decreasing, has limited the operational adoption of these approaches to large forest management groups, such as government bodies and companies overseeing the administration of large plantation forests. This has meant that individuals or smaller groups, such as local councils, have been left to continue their proximal forest inventory acquisition using conventional manual techniques. Low-cost remote sensing approaches that can accurately capture the 3D structure of trees, are therefore beneficial to such groups. It is this that has urged the exploration of proximal low-cost three dimensional (LC3D) remote sensing approaches, the most established among these being structure-from-motion (SfM) photogrammetry [14]. Previous studies have shown both terrestrial and airborne SfM approaches are able to provide accurate estimates of forest structure [15,16,17]. However, the expertise required to achieve accurate measurements, through strong camera geometry and accurate references of scale, as well as the high computational requirements needed to conduct SfM workflows at a plot scale, in a timely manner, have proved prohibitive to its operational adoption [14].

Recently, however, there has been an increase in the availability of LC3D technologies designed primarily for consumer applications. Most commonly, new LC3D devices incorporate colour and depth (RGB-D) sensors, that operate on either time-of-flight (ToF) or stereo triangulation approaches, to acquire range measurements. Devices utilising RGB-D sensors, typically also incorporate an inertial measurement unit (IMU), allowing for near real-time point cloud co-alignment using simultaneous localisation and mapping (SLAM) algorithms [18]. This access to SLAM processing workflows, makes these RGB-D devices similar to PLS in their mobile data acquisition. Furthermore, whilst available as standalone LC3D devices, RGB-D sensors are beginning to see integration into familiar consumer technologies, such as smartphones and tablets, increasing their accessibility both in terms of sensor procurement and familiarity of use. It is within these aspects that RGB-D devices may be a viable alternative over low-cost terrestrial photogrammetric approaches, as they can rapidly capture forest environments and the resultant point clouds can be visually assessed in near real time [19].

Current studies have largely focused on using active projection ToF RGB-D sensors for acquiring structural measurements of forest environments. One of the first ToF RGB-D sensors integrated into smartphones, was the Google Tango. This technology was utilised by Tomaštík et al. [20], who captured forest plots to extract stem diameter at breast height (DBH) and location, whilst investigating different SLAM capture paths. The authors found that capture approaches that do not repeatedly view the same stem, achieved accurate estimates of stem DBH and reduced the root-mean-square error (RMSE) (RMSE = 1.83 cm). The authors also found that, revisiting stems throughout the capture appeared to improve the accuracy of tree location estimates (stem position RMSE = 0.2 m), however, this resulted in increased DBH measurement error. In Hyyppä et al. [21], the authors also investigated the Google Tango technology, in comparison to the standalone Microsoft Kinect V1 active stereo RGB-D device, to measure DBH (Tango RMSE = 0.73 cm, Kinect V1 RMSE = 1.9 cm). As opposed to a continuous capture approach, trees were only measured individually, as the authors found that when attempting to capture more than one stem with either device, their performance became inconsistent, due to misalignment of depth frames caused by SLAM positional drift and incidental duplicate stem observations. We previously explored the use of the latest generation of the Microsoft Kinect Series, the Azure Kinect, a ToF RGB-D device. In an attempt to remove the point cloud misalignment error caused by SLAM drift, we used single depth images to estimate DBH. However, this approach was not as accurate as complete stem captures due to single stem perspectives not accounting for basal deformations within measured stems (RMSE = 8.43 cm) [22]. Recent studies have focused on the ToF RGB-D sensor integrated into the Apple iPad 2020/2021/2022 Pro and iPhone 13/14 Pro devices. The authors in Mokroš et al. [19], compared the Apple RGB-D sensor to the handheld PLS, stereo-camera SfM and TLS, for estimating stem diameter and location in forest plots. They found that, of the technologies utilised, the Apple RGB-D device provided the closest estimates of DBH compared to the TLS measurements (RGB-D RMSE = 3.14 cm, TLS RMSE = 1.45 cm). In Gollob et al. [23], they also explored the Apple RGB-D device and a series of different processing applications, in comparison to handheld PLS in forest plots. Using the optimal sensor application, the authors achieved similar DBH accuracy (RMSE = 3.13 cm). However, in this case, the Apple RGB-D device was outperformed by the handheld PLS, even though it was the same sensor used by Mokroš et al. [19] (GeoSLAM ZEB Horizon) (DBH RMSE = 1.59 cm). These Apple RGB-D technologies have also been applied in urban forest environments. Similar to the aforementioned studies in native forests, when measuring urban stem DBH, Wang et al. [24] found their optimal capture approach (individual stems) achieved an RMSE of 2.78 cm. Furthermore, the authors of Bobrowski et al. [25] found measurements of basal trunk flare derived with the Apple iPad Pro 2020, comparable to those captured with TLS (iPad RMSE = 8.7 cm, TLS RMSE = 7 cm). Whilst these previous studies have provided an assessment of the performance of individual RGB-D devices, few have provided a direct comparison between the technologies. It is therefore difficult to draw direct comparisons between RGB-D devices, as depth camera specifications, SLAM algorithms, capture approach, and environmental complexity all have an effect on overall performance.

Because of the limited range of these active projection RGB-D sensors (≤5.5 m under optimal conditions), stem structural measurements are restricted to those that can be acquired within this distance. Whilst passive triangulation RGB-D sensors may provide an extended measurement range (≤15 m), to our knowledge only the authors of Tran et al. [26] have explored these sensors for forest measurement, with other proximal passive remote sensing studies preferring alternative photogrammetric approaches [15,27,28,29,30]. Using single depth frames from the Intel Realsense D455 RGB-D device, Tran et al. [26] found measurements of stem DBH and curve, to a height of 2.9 m, comparable to SfM and TLS approaches. However, stem measurements at heights above this were difficult, due to sensor field of view (FoV) and occlusion from canopy vegetation.

An additional challenge with RGB-D devices, is that even though a sensor may be able to record depth frames, there is no guarantee that it will reconstruct an environment using SLAM, due to insufficient feature matches within the device’s capture path. This is because forest environments are one of the most difficult settings in which to conduct spatial mapping; the homogeneous texture and colour of features, as well as the presence of complex geometry with soft edges, results in issues with object identification and loop closure for drift correction [31]. Previous studies that have explored the application of RGB-D devices, have largely utilised them in open forest environments or urban settings. This is not consistent with the composition of native forest structures found within southeastern Australia. The close proximity of vegetation and irregular vertical growth pattern of eucalyptus species, increases the likelihood of repeat recordings of stems. This may result in stem duplication or deformation, due to misalignment error as a product of SLAM positional drift, when capturing stand information at a plot scale. Furthermore, the sparse canopy structure found within native eucalyptus forests, promotes the growth of understory vegetation elements around lower stem areas. The complex structure, soft edges, and obscuring nature of this vegetation has the potential to disrupt SLAM positional tracking and spatial mapping.

Within this context, the aim of this study is to: (1) investigate the effect of incidental stem captures, due to stem density, on DBH measurements acquired with low-cost RGB-D devices, (2) investigate the effect of understory vegetative elements, <1.3 m above ground height (AGH), within the capture range of RGB-D sensors, on the accuracy of DBH measurements captured with these devices, and (3) provide an inter-comparison between a selection of current RGB-D technologies and their associated SLAM algorithms, for acquiring basal stem measurements in urban and native southeastern Australian forest environments.

## 2. Materials and Methods

### 2.1. Study Area

Two study areas were selected for this paper, in order to provide a range of stem proximities and understory vegetation densities, to assess the effect these environmental factors may have on RGB-D device performance. Royal Park, an urban park located in Parkville, Victoria, in Melbourne CBD, was used as site one (S1). This park covers over 170 hectares and contains individual stems surrounded by maintained lawns, managed garden beds, and smaller areas of eucalyptus forest. Vegetation present within the park is primarily native to Australia, with the dominant captured species consisting of Eucalyptus Ironbark (*Eucalyptus sideroxylon*), River Red Gum (*Eucalyptus camaldulensis*), and Yellow Gum (*Eucalyptus leucoxylon*). S1 was used to assess the effect of stem proximity, and incidental duplicate captures, on the performance of a selection of RGB-D devices when estimating stem DBH measurements. A total of seven irregularly shaped plots, ranging between approximately 80 m and 590 m, and containing between 3 and 14 stems, were established, where the effect of stem proximity could be observed. Plots were captured until a range of stem diameters and distances between stems was achieved.

**Figure 1:**
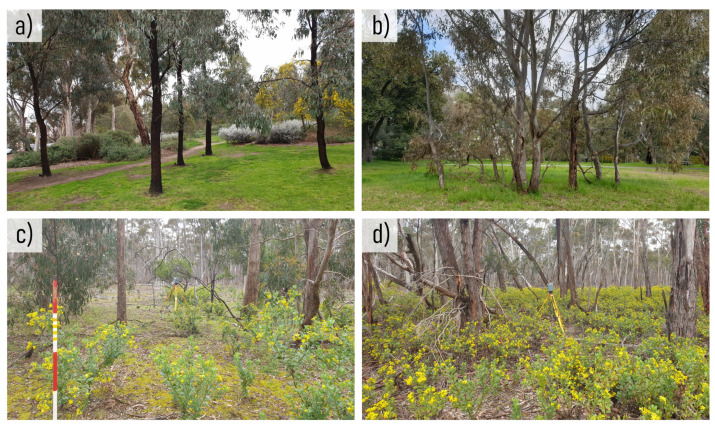
Examples of characteristic vegetation within each site environment. (**a**) Less dense stems at S1, (**b**) more dense stems at S1, (**c**) less dense surrounding understory vegetation at S2, and (**d**) more dense surrounding understory vegetation at S2.

The second site, site two (S2), was located within the You Yangs National Park, located approximately 55 km southwest of Melbourne CBD in Victoria, Australia. The national park consists of a series of granite ridges and surrounding native woodlands, with a maximum canopy height of approximately 15 m. Present throughout the plots is invasive Boneseed growth (*Chrysanthemoides monilifera*), that provides elevated soft edged vegetation with broad leaves. This soft edged understory vegetation surrounding stems, could be used to assess the effect that it may have on the performance of RGB-D devices. At S2, four sampling plots, 22.56 m in diameter, and containing between 9 and 25 stems, were established, to match the size of field plots used within the Victorian Forest Monitoring Program [32]. The information in Table 1 provides a summary of tree characteristics, and Figure 1 depicts examples of vegetation composition across both sites.

### 2.2. RGB-D Devices

Three RGB-D devices were selected, to assess the effect that stem density and understory vegetation elements may have on the accuracy of basal measurements derived from these technologies. The RGB-D devices used within this study were: (1) the Microsoft Azure Kinect (henceforth Kinect) (Microsoft Corp., Redmond, WA, USA) [33], (2) the Apple iPad Pro 2020 (henceforth iPad) (Apple Inc., Cupertino, CA, USA) [34], and (3) the Stereolabs Zed 2 (henceforth Zed) (Stereolabs Inc., San Francisco, CA, USA) [35]. These devices were selected as they represent both RGB-D sensors integrated into consumer technologies (iPad), and those that require integration with a CPU, data storage, and an external power source (Kinect and Zed) but offer greater flexibility in surrounding capture and processing settings. These devices also demonstrate RGB-D technologies with bespoke SLAM algorithms designed for the device (iPad = Apple ARKit; Zed = Zed Fusion), or those that require integration with an open source algorithm (Kinect = RTAB-Map). Lastly, they provide an example of two common depth sensing approaches, in ToF RGB-D (iPad and Kinect) and passive stereo (PS) triangulation RGB-D (Zed).

The following sections present an outline of the capture and processing approaches used to acquire 3D representations of stem structures with these RGB-D devices and their associated SLAM algorithms.

### 2.3. RGB-D Data Capture

**Figure 2:**
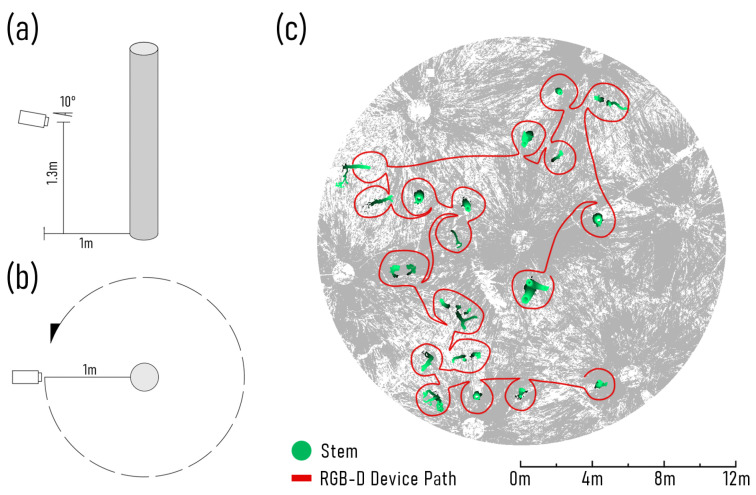
(**a**) Depicts how colour and depth (RGB-D) devices were held relative to each stem during capture, (**b**) shows how each stem was circumnavigated using both the individual stem and continuous capture approaches, and (**c**) provides an example of the path used to move the RGB-D devices through a plot at S2, native woodland, using the continuous capture approach.

Across both S1 and S2, the capture approach to acquire depth image streams was kept consistent with all three RGB-D devices. At both sites, a continuous capture approach was used to acquire depth image streams. After each plot had been established, a starting stem was identified, based on its location towards the edge of a group of stems or plot. Holding the RGB-D device at a height of approximately 1.3 m—angled down slightly to keep the ground within view (approximately 10${}^{\circ }$), and facing towards the stem at a distance of 1 m—the depth image capture was started. The operator then moved at a slow walking pace (approximately 0.7 m per second) around the stem, until it had been captured from all angles (Figure 2). Care was taken to ensure that object surfaces did not pass within the self-occlusion range of each RGB-D sensor (ToF ≤ 0.3 m, PS ≤ 0.5 m), to reduce the number of dropped depth frames that may introduce SLAM error. Once the first stem had been completely captured, the operator slowly walked the device to the next closest stem, making sure that the slight downwards facing angle of the sensor was maintained and attempting to avoid any abrupt movements. This downward angle ensures that ground surface points continued to be captured, as depth frames absent of depth measurements may also introduce SLAM error, due to a lack of feature matches. This process was repeated until all stems within the target area had been captured.

In addition to the continuous capture approach, an individual stem capture approach was also conducted. The justification behind this was twofold. Primarily, this was to ensure that all stems within each plot were represented, even if the SLAM algorithm failed to co-align depth frames, by losing its spatial position estimate due to complex understory vegetation, when moving between stems. Furthermore, it also provided another point of reference for assessing the effect of duplicate stem captures, that may be present with a continuous capture approach. For individual stem captures, the same approach was used regarding sensor height, distance, and orientation in relation to the target stems, the only point of difference, being that depth image capture was stopped when moving between stems and a new capture begun for each tree within the plot.

Due to active projection RGB-D devices experiencing increased noise and reduced capture range under strong ambient lighting conditions [22], depth recordings with RGB-D devices were conducted under partial cloud (S1) and overcast (S2) conditions.

#### 2.3.1. Azure Kinect Data Capture Parameters

The depth sensor included within the Kinect device, acquires range measurements on the basis of active projection ToF. Based on our previous work, we determined the optimal capture parameters for the Azure Kinect sensor in an outdoor environment, to be a narrow FoV with binned depth pixels (resolution = 320 × 288 pixels, FoV = 75${}^{\circ }$× 65${}^{\circ }$) [22]. Using the K4Aviewer software, provided by Microsoft as part of the Azure Kinect development kit, and the aforementioned capture settings, depth images and associated inertial data from the device’s IMU were recorded, at intervals of 30 frames per second. Once RGB-D recordings had been captured at each site with the Kinect, the software RTAB-Map was used to generate 3D point clouds of each capture path [36]. RTAB-Map provides a graph-based SLAM solution, that can be applied to a range of supported RGB-D devices, to create a spatial map of the recorded environment, composed from the registered depth frames. Within RTAB-Map, the maximum depth range was limited to 3 m, and IMU recordings were filtered and smoothed using the provided Magdwick algorithm. The distance of 3 m was selected for depth range based on initial exploration with RTAB-Map and previous research with the Kinect device [22]. RGB camera frames were used, as opposed to near infrared (NIR) frames, for feature detection, due to sufficient lighting during the capture process. The resultant spatial maps were then exported as point clouds for assessment.

#### 2.3.2. iPad Pro Data Capture Parameters

The RGB-D sensor incorporated within the iPad is a ToF sensor, and therefore operates with the same depth acquisition principals as the RGB-D sensor within the Kinect. Due to being integrated into a tablet, there are a series of applications available to the iPad device, that can be used to capture, pre-process and visualise 3D point clouds. In this study, we used the SiteScape application (Version 1.3) [37] (SiteScape Inc., New York, NY, USA). This application was selected based on the previous study by Gollob et al. [23], who assessed a series of available applications for the iPad device, with their results suggesting that the SiteScape application was the most accurate when acquiring stem DBH measurements. Furthermore, in the version of the software that we used, the iPad display overlaid the captured point cloud on top of real-time imagery of the scene, captured with the RGB camera, providing augmented reality feedback to the operator. This made it easier to identify target stems in structurally complex forest environments. Based on the findings of Gollob et al. [23], and our initial exploration with the application, the following capture settings within the software were used: scan mode = maximum density, point density = high, and point size = low.

A major point of difference between the iPad and the two standalone Kinect and Zed RGB-D devices, is that depth measurements were automatically aligned and visualised as a point cloud on the tablet’s screen, allowing for visual assessments of point cloud quality to be conducted in near real time. Furthermore, where the standalone sensors could capture indefinitely, until they run out of battery or storage space, the iPad only recorded for a certain number of points (based on application capture parameters and data size) before automatically stopping. In this case, a new recording was immediately started where the last ended, and the scan path finished when conducting the continuous capture approaches. As SiteScape is able to hold position within the arbitrary coordinate space established within the first scan, the resultant point clouds from subsequent scans were easily checked for alignment and fused afterwards within CloudCompare (CloudCompare, ver. 2.11.3, Paris, France) [38]. Due to limited control surrounding depth measurements and the SLAM process with the iPad, the maximum depth measurement range of the sensor during spatial mapping could not be reduced. Therefore, all depth measurements, up to a maximum sensor distance of approximately 5.5 m, were considered.

#### 2.3.3. Zed2 Data Capture Parameters

The Zed RGB-D sensor captures depth frames on the basis of passive triangulation. This approach estimates the distance between the sensor and objects based on feature matches between pixels and the baseline distance between the optical centre of each camera. Stereo image streams were acquired using the ZEDexplorer application provided by Stereolabs. Within the software, the default camera configuration was used, except for the following settings: image resolution = 1920 × 1080, frames per second = 30, and image enhancement on. Stereo image recordings captured from both sites were then loaded into ZedFusion. This application takes the stereo image stream and the sensor calibration file provided with, and unique to, each Zed device and creates depth frames from the paired stereo images. These stereo images are then used to create a spatial map of the captured environment using the in-built SLAM algorithm included within ZedFusion. When processing the Zed spatial map, depth measurements were limited to 3.5 m. This was done as, whilst ZedFusion allows for range measurements up to 10 m during the SLAM process, it was found, during initial exploration, that when processing with measurement ranges greater than this, there was an increased likelihood of duplicate stem representations in the resultant point cloud. The spatial map of each recording was then exported as a point cloud, for further analysis.

### 2.4. TLS Data Capture

Point clouds captured with a Trimble TX8 TLS, were used as a reference to validate the performance of the RGB-D devices. Whilst there are still observational errors within TLS datasets, it is considered to be the benchmark sensor that emerging terrestrial 3D remote sensing technologies are compared to within forested environments [4,39]. The TX8, costing approximately USD 70,000, operates on the basis of ToF, emitting pulses of NIR EMR and using the travel time for a pulse to reflect off a surface within the environment and return to the sensor to provide an accurate estimate of the distance to that object [40]. Based on this range estimate and the angle at which the pulse was emitted, the location of the surface can then be determined relative to the sensor.

At S1, scan stations were placed at 10 m intervals, until all faces of each stem within the target area were captured. For S2, a seven scan approach was used, to acquire point clouds of each plot. This incorporated a single scan at the centre of the plot and six scans towards the exterior boundary of the plot, approximately 10 m from the plot centre, located at 60${}^{\circ }$ intervals from the centre scan. TLS point clouds were processed using the Trimble Realworks 11.1 software (Trimble Inc., Sunnyvale, CA, USA), native to the TX8. Scans were automatically co-aligned using the cloud based registration tool. After automatic registration the location of each scan station was visually assessed and manually adjusted where considered necessary, before refining the registration until an acceptable level of error was achieved (<10 mm). Registered point clouds were then exported for the extraction of metrics.

### 2.5. Point Cloud Processing and Stem Diameter Estimation

Point clouds derived from both the RGB-D sensors and their associated SLAM algorithms, and the TLS, were visually assessed and edited for noise using a nearest neighbour approach, within CloudCompare. TLS point clouds were then cropped to the extent of each plot. This was done manually for the irregular shaped plots captured at S1, making sure to include all stems captured with RGB-D devices, and automatically, using an in-house Python library and a 12 m radial crop, based on the central TLS scan location, for the plots captured at S2. This step was not required for the RGB-D point clouds, as the limited range of the sensors and SLAM depth threshold, meant that only the stems of interest and directly surrounding environmental features were captured.

The cleaned point clouds were then imported into 3DForest (National Land Survey Service, ver. 0.52, Helsinki, Finland), an open source software for visualising and measuring common stem and crown attributes from point clouds of forest structures [41]. Ground points were first segmented from each point cloud using an octree approach, with a 10 cm^3^ voxel resolution. This approach classifies the points within the lowest filled voxels as the ground layer and all other points as vegetation. Individual stems are then identified from the vegetation points using the automatic segmentation tool, as described in Avery and Burkhart [5]. When conducting automatic segmentation, the following parameters where used: voxel size = 5 cm^3^, descriptor threshold value = 70%, iterations = 5, voxels in element = 100, distance from terrain = 0.5 m, and descriptor variable = principal component analysis. Stems were then visually assessed for correct segmentation, left over vegetation points were removed, and segmentation was repeated on still grouped stems, using a finer 2 cm^3^ resolution. Stem base position was then estimated as the median coordinate for all stem points above the classified ground points and 1 m AGH. These points were considered to account for any stems that had significant basal sweep, that may otherwise estimate their stem location away from their DBH measurement location. For some stems captured with RGB-D devices at S2, there was poor representation of ground features through dense understory vegetation. In these cases, the ground height was considered to be the height of the lowest 98th percentile of points within the cloud. Stem DBH values were then estimated using the ground height at stem location for the reference and a least squares regression (LSR) fit at 1.3 m AGH. This process was repeated with both continuous and single stem captures from all devices (RGB-D and TLS) across both sites.

### 2.6. Stem Density and Soft Edged Vegetation Estimation

Stem proximity and understory vegetation volume was estimated using the TLS reference point cloud datasets. At S1, proximity values were assigned to each tree using the distance to the next closest stem within the target plot. The rationale behind this metric, is that a tree with a shorter distance to another target stem will have an increased likelihood of being accidentally captured multiple times, due to being within the depth measurement range of an RGB-D device whilst another stem is the target. Therefore, this means that whilst the surrounding stems are being captured, there is an increased chance of a duplicate capture occurring that, due to SLAM positional drift, may result in misalignment error. Due to the different maximum depth ranges of each RGB-D device, this duplicate capture distance varied between each instrument.

For S2, estimates of understory vegetation volume (≤1.3 m AGH) surrounding each stem was calculated. This was conducted for each stem, by extracting circular subplots (2 m radius for Kinect assessment, 2.5 m radius for Zed assessment, and 4.5 m for iPad assessment) for each stem within each plot. The radius of these subplots was determined by the depth measurement range of each RGB-D device, e.g., as the Kinect depth measurements were limited to 3 m and the sensor was held 1 m away from the face of the stem, the 2 m radial subplot represents the area within range of the sensor. Each subplot was then cropped further, to points ≤1.3 m AGH, this was to consider all understory vegetation below RGB-D sensor height. A cloth simulation filter was then used to segment and remove ground points [42]. The parameters used with this cloth simulation filter were: grid resolution = 0.24 m, class threshold = 0.03 m, time step = 0.6 s, iterations = 1000, and rigidity = 3. The target stem was then extracted from each subplot, and soft vegetation volume calculated. This approach involved first creating a voxel space for each subplot, composed of 10 mm^3^ cells. As the upper layer of the understory vegetation occludes elements beneath it, volume was calculated using the highest point return within each voxel column and considering all space beneath it as filled. Vegetation volume was then calculated as the sum of all filled voxels.

### 2.7. Stem Diameter Measurement Analysis

Stem DBH estimates derived with the RGB-D devices, were compared to estimates derived with the TLS, across both sites. To compare performance between the RGB-D devices, scatter plots comparing the DBH acquired with each RGB-D device to TLS estimates were generated, and the RMSE and root-mean-square percentage error (RMSPE) for each RGB-D device at S1 and S2 were calculated. Plots were also generated comparing the stem proximity value (S1) or understory vegetation volume (S2), to stem DBH RMSE. Welch’s *t*-test was used to assess if there was a significant different within DBH measurement error between stems within the duplicate capture range of each RGB-D device captured at S1, with the continuous approach and the individual approach (absent of incidental duplicate captures caused by stem proximity). A Pearson’s correlation coefficient statistical test was conducted to check for a significant relationship between understory vegetation and the DBH error observed at each stem, with each RGB-D device, at S2. In addition, RGB-D point clouds, captured at both sites, were visually assessed for misalignment error or incomplete stem representation, in comparison to point clouds derived with the TLS. For the Kinect and Zed devices, the raw depth imagery was also visually inspected.

## 3. Results

### 3.1. Colour and Depth Sensor Performance

**Figure 3:**
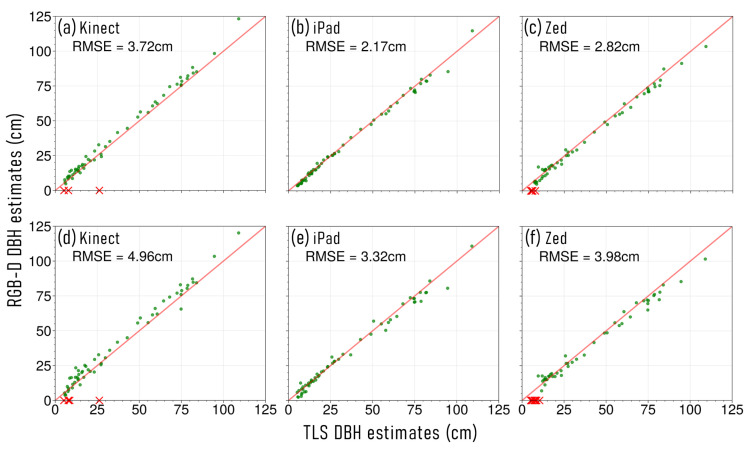
Stem diameter at breast height (DBH) estimates captured at site 1 (S1), in urban parkland, using colour and depth (RGB-D) sensors, and compared to terrestrial laser scanning (TLS) measurements. (**a**) Microsoft Azure Kinect individual stem capture, (**b**) Apple iPad Pro 2020 individual stem capture, (**c**) Stereolabs Zed 2 individual stem capture, (**d**) Microsoft Azure Kinect continuous capture, (**e**) Apple iPad Pro 2020 continuous capture, and (**f**) Stereolabs Zed2 continuous capture. The red × symbols along the *x*-axis represent the measured TLS DBH of stems that were unable to be captured using the respective RGB-D device.

**Figure 4:**
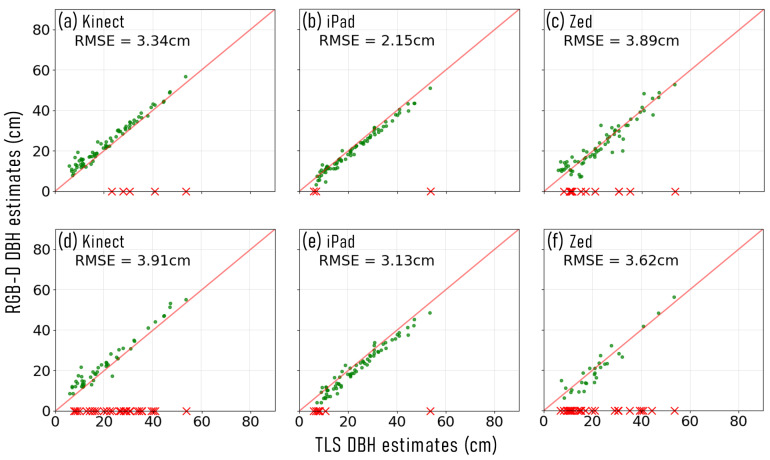
Stem diameter at breast height (DBH) estimates captured at site 2 (S1), in native woodland, using colour and depth (RGB-D) sensors, and compared to terrestrial laser scanning (TLS) measurements. (**a**) Microsoft Azure Kinect individual stem capture, (**b**) Apple iPad Pro 2020 individual stem capture, (**c**) Stereolabs Zed 2 individual stem capture, (**d**) Microsoft Azure Kinect continuous capture, (**e**) Apple iPad Pro 2020 continuous capture, and (**f**) Stereolabs Zed2 continuous capture. The red × symbols along the *x*-axis represent the measured TLS DBH of stems that were unable to be captured using the respective RGB-D device.

The number of stems successfully reconstructed at each site, with each RGB-D device and approach, and the DBH measurement error, is presented in Table 2. Across both sites, and using both approaches, the iPad achieved the lowest RMSE when estimating DBH, when compared with TLS measurements (Figure 3 and Figure 4). At S1, the Zed was observed to have the next lowest RMSE with both capture approaches. However, at S2, when using the individual stem capture approach, the Kinect device achieved a lower comparative DBH RMSE. Furthermore, across both sites and capture approaches, the Kinect was able to successfully reconstruct point clouds of more stems than the Zed device.

DBH estimates acquired across both sites, that used an individual stem capture approach, resulted in lower RMSE. The exception to this, was for the Zed device at S2 (Figure 4c,f). However, although the Zed reported lower DBH RMSE when using the continuous capture approach at S2, it was only able to represent 28/79 stems, compared to the 66/79 reconstructed using the individual capture approach. Using the individual stem capture approach also allowed an increased number of stems to be successfully described with the Kinect and Zed devices, across both sites (Table 2). Whilst the iPad also captured more stems at S2 with the individual capture approach, it was able to capture point cloud representations of all stems at S1 with both data acquisition methods.

For both sites, the Kinect device was observed to overestimate DBH measurements when using both capture approaches, when compared to TLS values (Table 2). Conversely, the iPad and Zed devices underestimated stem DBH measurements across both sites. The exception to this was at S2 when using the Zed device and the individual stem capture approach, where there was observed to be a slight positive bias.

### 3.2. Effect of Stem Proximity on RGB-D DBH Estimates

**Figure 5:**
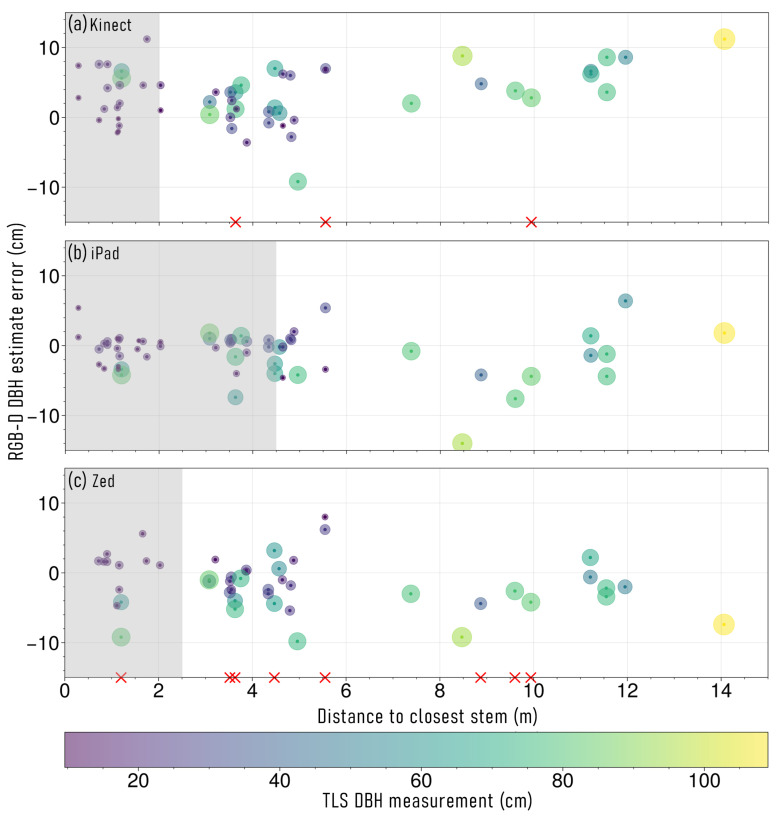
Stem diameter at breast height (DBH) measurement error (cm) derived from colour and depth (RGB-D) sensors, and the distance from target stem to the next closest stem. (**a**) Microsoft Azure Kinect, (**b**) Apple iPad Pro 2020, and (**c**) Stereolabs Zed 2. The symbol size and colour around points represents the DBH of each stem measured with terrestrial laser scanning (TLS). The red × symbols on the *x*-axis represent trees that were unable to be captured using the respective RGB-D sensor and the distance to the next closest stem within their plot. The grey shaded section on each plot represents the range in which duplicate stems could occur for each RGB-D sensor.

DBH measurement error, present within RGB-D observations, in relation to stem proximity, is shown in Figure 5. When visually assessing point clouds captured at S1, all three RGB-D devices were observed to experience some form of misalignment error, as a result of duplicate stem captures and positional drift, where a stem fell within the observation range of another stem for that specific RGB-D device. Despite this misalignment error however, no significant difference in RMSE was observed for stems within the duplicate capture range of another tree for the individual and continuous capture sets (iPad t(36) = 1.11, *p* = 0.27; Kinect t(16) = 1.44, *p* = 0.16; Zed t(10) = 0.27, *p* = 0.79).

Using the iPad, of the trees captured at S1, 40 stems fell within the 4.5 m duplicate capture range of this device (Figure 5b). The RMSE of these 40 stems, when using the continuous capture approach, was 2.32 cm, with a mean observed TLS DBH of 26 cm (RMSPE = 8.9%). The remaining stems that did not experience duplicate capture, had a DBH RMSE of 4.72 cm and a mean DBH of 52.6 cm (*n* = 20, RMSPE = 9%). Using the Zed device, 23 captured stems were within the 2.5 m duplicate capture range (Figure 5c). Of these stems, 11 failed to capture with the Zed and the remaining 12 had a DBH RMSE of 3.9 cm, with a mean DBH of 23.5 cm (RMSPE = 16.6%). The stems outside of this range, had a DBH RMSE of 4.01 cm, with a mean DBH of 48.9 cm (*n* = 35, RMSPE = 8.2%). The Kinect had a total of 21 stems within the 2 m duplicate capture range of the device. Three of these stems failed to capture with the Kinect device, with the remaining 18 having a DBH RMSE of 5 cm, with a mean DBH of 17.7 cm (RMSPE = 28.2%). The rest of the stems outside the duplicate capture range of the Kinect, had an RMSE of 4.93 cm and a mean DBH of 45.4 cm (*n* = 38, RMSPE = 10.9%).

### 3.3. Effect of Understory Vegetation on RGB-D DBH Estimates

The plots established at S2, native woodland, were used to assess the effect of understory vegetation (≤1.3 m) on the DBH estimates derived by the RGB-D devices. When using the continuous capture approach, the iPad was the only device that was able to maintain SLAM spatial tracking within all four plots. When using the Kinect device, 25/31 (81%) failed stem captures were due to a loss of spatial tracking within the SLAM algorithm, that resulted in depth images failing to co-align. This loss of spatial positioning occurred within plot one (understory cover = 56.6% and volume = 43.5%) and plot four (understory cover = 35.7% and volume = 27.4%). The Zed device experienced similar issues, with 47/51 (92%) failed captures being due to a loss of positional estimate within the SLAM process. Both the Kinect and Zed devices were only able to maintain SLAM spatial tracking for the entirety of plots two (understory cover = 42.3% and volume = 32.6%) and three (understory cover = 17.4% and volume = 13.4%).

**Figure 6:**
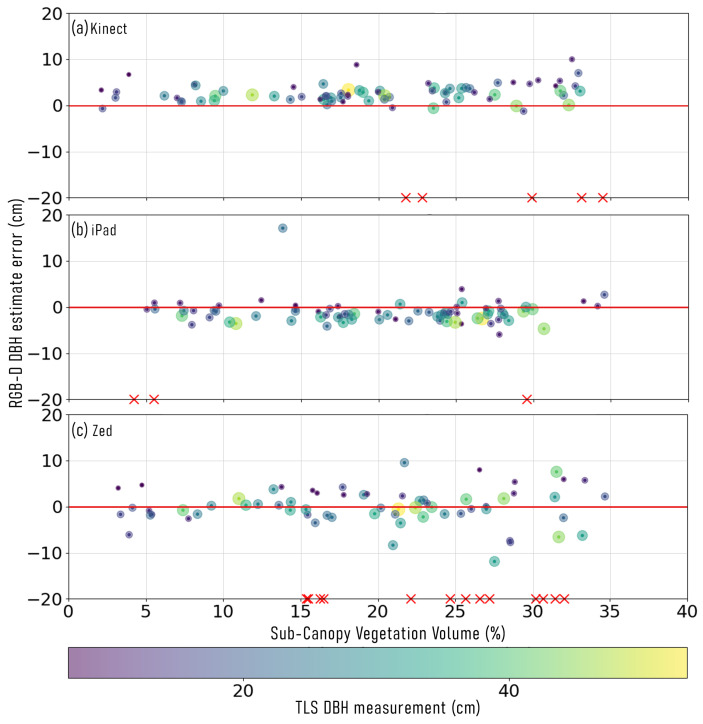
Stem diameter at breast height (DBH) measurement error (cm) derived from colour and depth (RGB-D) sensors, and the volume of understory vegetation surrounding each stem within RGB-D device range and below device height (1.3 m). (**a**) Microsoft Azure Kinect, (**b**) Apple iPad Pro 2020, and (**c**) Stereolabs Zed 2. The symbol size and colour around points represents the DBH of each stem measured with terrestrial laser scanning (TLS). The red × symbols on the x-axis represent trees that were unable to be captured using the respective RGB-D sensor and the volume of understory vegetation within the capture range of that stem.

When the individual stem capture approach was used, all devices were able to reconstruct a greater number of stems, compared to when using the continuous capture approach (Table 2). For DBH values derived from the Kinect device (Figure 6a), there was observed to be a low, but significant, correlation between DBH error and surrounding understory vegetation volume (r(72) = 0.24, *p* = 0.04). Conversely, no significant relationship was observed between DBH measurement error and understory vegetation volume within the measurement range of successfully captured stems when using the iPad and Zed devices (iPad *p* = 0.55, Figure 6b; Zed *p* = 0.86, Figure 6c). Furthermore, the iPad and Zed devices both presented very low correlation between DBH error and understory vegetation volume (iPad r(72) = −0.07, Zed r(64) = 0.02).

## 4. Discussion

This study investigated the impact of stem proximity and surrounding sub-canopy vegetation (≤1.3 m AGH) on measured stem DBH, when using contemporary consumer grade RGB-D devices, providing an accuracy performance for each. This was done by analysing data collected across urban parkland, to assess stem proximity and incidental duplicate captures, and native southeastern Australian woodland, to assess sub-canopy vegetation density, using individual stem and continuous capture approaches, and comparing them to TLS. The RGB-D devices used within this study, were either standalone (Kinect and Zed) or integrated into consumer devices (iPad), and provided examples of common depth sensing technologies, in the form of active projection ToF (Kinect and iPad) or passive triangulation (Zed). Each RGB-D device had an associated SLAM algorithm, used to register individual depth frames together and create point cloud representations of stem structures. However, as a result of this, it is difficult to decouple error from that which originates within the depth imagery or the SLAM registration process. The following sections provide discussion points surrounding sensor performance, the effect of the investigated environmental factors, RGB-D sensor limitations, and future research directions for this low-cost technology.

### 4.1. Sensor Performance

Sensor performance was assessed for tree allometry for the Kinect, iPad, and Zed. Of the three RGB-D devices, the iPad was observed to have the best performance, presenting the lowest DBH RMSE and successfully resolving the greatest number of stems, across all sites and using both data capture approaches. The results for the iPad DBH RMSE derived from the continuous capture approach (S1: urban parkland DBH RMSE = 3.32 cm, S2: native woodland DBH RMSE = 3.13 cm), correlate with those reported within previous studies by Gollob et al. [23] and Mokroš et al. [19] who, when using the iPad in open forest environments, reported DBH RMSEs of 3.13 cm and 3.14 cm, respectively, from similar capture approaches.

**Figure 7:**
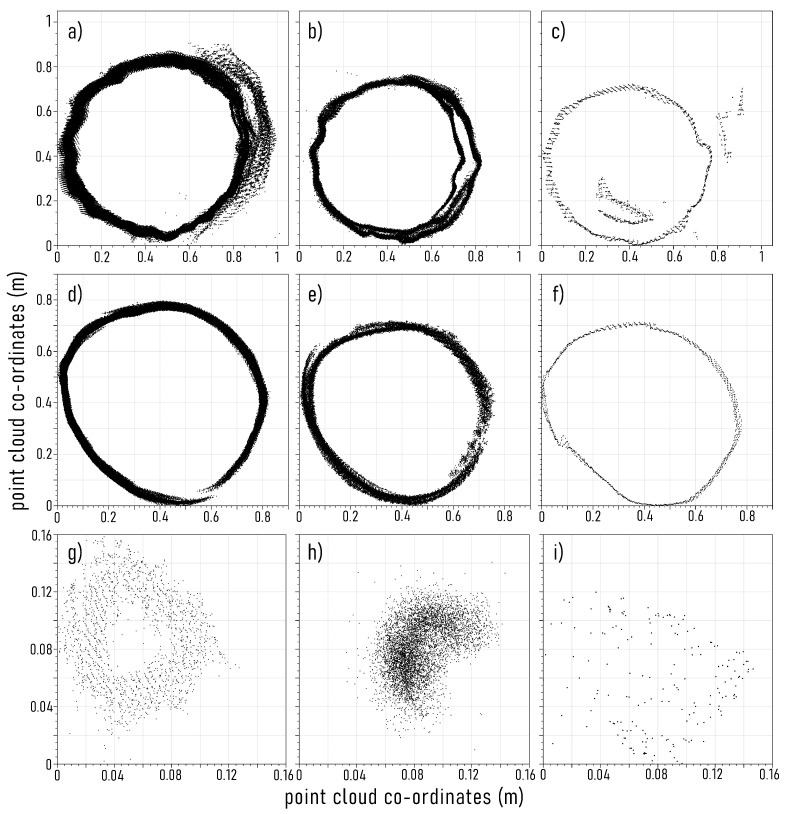
Examples of depth image misalignment error, from the continuous stem capture approach, as a result of incidental duplicate captures, with (**a**) Microsoft Kinect, (**b**) Apple iPad, and (**c**) Stereolabs Zed 2 colour and depth (RGB-D) devices (TLS DBH = 74.8 cm); and examples of depth image misalignment error as a result of simultaneous localisation and mapping (SLAM) drift with (**d**) Microsoft Kinect, (**e**) Apple iPad, and (**f**) Stereolabs Zed 2 colour and depth (RGB-D) devices (TLS DBH = 68 cm). While stems ≤15 cm can be represented, they are often poorly so (TLS DBH = 7.5 cm): (**g**) Microsoft Kinect, (**h**) Apple iPad, and (**i**) Stereolabs Zed 2.

Within the urban parkland environment, the stems that failed to be represented with the Kinect and Zed devices all had TLS DBH values ≤ 8.6 cm and ≤ 10.4 cm, respectively. The exception to this, was a single tree reconstructed by the Kinect device that, whilst having a DBH value of 26.2 cm, had low branches, resulting in foliage around sensor height, that prevented an unobscured and complete view of the stem. This obfuscation of stems was also observed within the native woodland plots. In addition, large trees (DBH ≥ 20 cm) were sometimes unresolved, due to a loss of SLAM position when using the continuous capture approach. Where stems with DBH values < 15 cm were resolved by any of the three RGB-D devices, the representation of overall stem structure was poor, due to either noise off the face of the stem, sparse point density, or only once face of the stem being characterised (Figure 7g–i). These results concur with findings from previous studies, where the representation of stems with DBH ≤ 10 cm to ≤15 cm, are inconsistently resolved when using RGB-D devices [19,23,25].

For all of the assessed RGB-D devices, the individual stem capture approach achieved a lower DBH RMSE. This agrees with the authors of Bobrowski et al. [25], who, when using the iPad device, found that there was a significant difference in stem perimeter at breast height (PBH) estimates between scanned groups of three and six trees, with the scanned groups of three stems resulting in lower measurement errors. Individual tree captures also resulted in an increase in the number of stems that were successfully represented by the Kinect and Zed across both sites and the iPad in native woodland plots. This increase in representation was more evident within native woodland, and was due to limitations within the Zed and Kinect SLAM algorithms. A complete loss of spatial position estimates during data acquisition, resulted in all trees subsequent to the loss of position to be missing in the final point cloud, due to depth frames no longer being aligned. While it is difficult to identify the precise cause, the positional estimate of the device being lost is likely due to the increased complexity of vegetation features within these native woodland plots, as opposed to the urban parkland environment. This understory vegetation obscures the RGB-D sensor’s view of the ground surface, that would otherwise act as a consistent plane of reference for the fusion of depth images; and the abundance of soft edged vegetation, comprising homogeneous colour and texture, complicates the identification of unique feature points.

The iPad was more resilient to this effect. Due to the black-box nature of the device’s SLAM algorithm, and limited access to raw depth imagery however, determining the direct cause of this is difficult. Based on the visual assessment of point clouds achieved with the iPad, it is speculated that there may be noise filtration of raw depth images as a pre-processing step, before being co-aligned with the Apple SLAM algorithm. Such a noise filter would remove much of the complex geometry of low canopy foliage and understory vegetation present within the native woodland plots, and would explain its limited representation within iPad point clouds. This proposition is further supported by the negative bias reported within stem DBH estimates derived from the iPad device, a likely outcome of the filter, which would remove the back scatter noise that is the result of mixed NIR reflectance depth pixels along the edge of stem objects. In contrast, the Kinect device operates on the same ToF RGB-D approach, but uses the raw depth images within the SLAM process, it is this backscatter edge effect that likely results in the positive bias present within DBH estimates acquired with this device. Although the iPad provided representations of hard surfaces of stem features, due to much of the softer vegetative material being absent from scans, this device may not be appropriate for describing vegetation other than large woody structures.

Whilst the single stem approach resulted in lower measurement error, it is important to note that capturing individual stems, as opposed to continuous capture, resulted in longer acquisition times. On average, capturing an 11.28 m radial plot, comprising between 15 and 27 stems, took between five and seven minutes. Comparatively, the single stem approach took between 15 and 18 min for the same plots. However, much of this time was spent stopping and starting sensor capture, and checking for successful output. Although not assessed in this study, the individual stem capture approach also limits the capture of plot-scale inventory metrics, such as stocking density and stem location. A potential solution to these issues may utilise a continuous depth stream capture, to reduce capture time and allow for assessment of plot-scale vegetation metrics, that can then be broken down into each individual stem capture for extraction of metrics.

### 4.2. Effect of Stem Proximity and Understory Vegetation

Previous studies have shown that prior generations of RGB-D devices have experienced substantial misalignment errors, resulting from continuous capture approaches, that cause repeat observations of tree structures [20,21]. Whilst recent studies have largely explored the Apple iPad RGB-D device for measurements of basal forest structure, these studies have been conducted in open forest environments [8,19,24,25,43]. This study sought to better understand the effect of duplicate stem observations on contemporary RGB-D devices and how they perform in native woodland environments with understory vegetation of varying complexity.

Although evidence of misalignment error due to incidental duplicate captures and SLAM positional drift was visible within the RGB-D point clouds (Figure 7), there was not observed to be a significant difference in stem DBH error when comparing estimates acquired with the continuous and individual stem capture approaches. It should be noted, that the smaller capture ranges of the Zed and Kinect devices, compared to the iPad, as well as their inability to consistently resolve stems with DBH ≤ 15 cm, led to smaller sample sizes and therefore reduced statistical power.

Based on the visual assessment of RGB-D point clouds, duplicate captures of stems resulted in points derived from incidental observations of stem faces being slightly misplaced, either off the face of points from the intended stem capture, or more often in the case of larger diameter stems, overlapping the deliberate observation. This slight misalignment, however, is further reduced when estimating stem DBH, as the LSR diameter estimation approach places a best fit circle through the mean distribution of points, effectively smoothing misalignment error. Slight misalignment error was also visible within point clouds of stems captured with the individual stem approach. This is because of SLAM drift whilst the operator circles around an individual stem, as well as accidental over observation of a stem as a result of capturing past the initial capture point on a stem face, resulting in re-observation. An example of this is presented within Figure 7d–f). Whilst care was taken to avoid this as much as possible, it is outside of operator control in the case of drift, and difficult to consistently avoid in the case of re-observation. However, this error was less than that observed within the continuous capture approach. The authors of Bobrowski et al. [25], found that multiple rotations around a stem increased the accuracy of tree PBH estimates when using the iPad device. However, they could not discern why this effect was observed. We speculate this may be due to overlapping observations of stem structure and SLAM drift reducing the negative bias associated with the stem DBH observations we observed with the iPad device. Regardless, it is evident that, where earlier iterations of RGB-D devices—such as the Google Tango and the Microsoft Kinect V1—struggled with the continuous capture of multiple stem structures, as suggested by Hyyppä et al. [21], the current generation of RGB-D devices were more successful when utilising a continuous plot-scale capture approach, particularly in open forest environments, such as the urban parkland investigated at S1. While contemporary RGB-D sensors contain improved imaging hardware compared to previous generations, it is likely that the smaller misalignment error resulting from incidental duplicate observations of stem faces, experienced by current RGB-D devices, is a result of advancements within SLAM algorithms, IMU accuracy, and computational power. However, as reported by Gollob et al. [23], RGB-D devices still struggle with actively revisiting stems that have previously been captured, resulting in larger misalignment errors. This is likely because the loop closure process, used by SLAM algorithms to reduce positional drift, is reliant on recognising the re-visitation of uniquely unidentified environmental features. Where RGB-D SLAM algorithms are designed primarily for use within man-made environments, this means that in forests with homogeneous colour and texture, as well as soft edged surfaces, this can be difficult.

While there was not observed to be a significant relationship between the volume of understory vegetation within the capture range of each RGB-D device and DBH error when capturing individual stems, the structural complexity of understory vegetation within the sensor’s view is likely what caused the SLAM algorithms used by the Kinect and Zed devices, to lose positional estimate within two of the plots. It is, however, difficult to quantify the exact environmental conditions that resulted in this error. The exception to this was with the Kinect sensor within one plot, where understory vegetation passed too close to the RGB-D sensor, resulting in self-occlusion and loss of depth imagery for five frames (one-sixth of a second). This was enough for the RTAB-Map SLAM algorithm to lose spatial positioning. The iPad was the most robust in this case, and as mentioned before, this is likely due to some form of noise filtration that takes place before the SLAM process, as much of the finer vegetation elements present within the TLS point clouds are absent from the final iPad point clouds. The black-box nature of the Apple API, makes it difficult to accurately identify what is occurring.

### 4.3. RGB-D Limitations and User Experience

One of the major benefits offered by RGB-D devices that have been incorporated into handheld devices, such as mobile phones and tablets, is that they present the opportunity to rapidly acquire and visualise point clouds in near real time. It is due to this visualisation of point cloud imagery, as well as the small form and light weight of the device, that the iPad offered the best user experience of the technologies assessed within this study. This increasing integration of RGB-D sensors within consumer devices, lowers the barrier of entry for acquisition of 3D representations of basal forest structures, both in terms of hardware accessibility and familiarity of use. However, whilst the extraction of simple and common forest inventory metrics is available with some applications, such as the *ForestScanner* application for Apple RGB-D devices [43], there are limitations with access to raw depth images or point cloud data, and further expertise is required for the processing and extraction of more complex metrics, such as basal taper and sweep.

While RGB-D devices share similarities with PLS, the FoV of the sensor is limited to directly in front of the device. This means that each stem has to be manually identified by the operator and circumnavigated. Whilst this is relatively straightforward in urban parkland, open forests, and managed plantation environments, it was found to be more difficult in the native woodland plots, where, in some situations, dense understory vegetation obstructed the visibility of target stem faces and inhibited the operator’s path around the stem. Furthermore, to acquire reliable inertial data, the device should be moved through the plot in a smooth and controlled manner—something that is difficult if the view of the ground is obstructed, uneven, or there are tripping hazards, such as fallen limbs, present within the plot. Because of this, the most reliable data capture approach in such environments is likely an individual stem capture approach, that also resulted in lower DBH RMSE. However, this capture approach foregoes the acquisition of other important plot-scale metrics, such as stem position, and can make it difficult to validate whether a captured stem located close to a plot boundary, should be considered within the inventory sample.

Beyond this, one of the greatest drawbacks associated with RGB-D devices is their capture range, that limits structural measurements to the lower basal areas of stems. Whilst this may be somewhat overcome by panning the device up stems to capture their elevated structure, it was found in initial exploration, that this may introduce duplicate captures if the device is panned back down the stem, and losing view of the ground plane may also introduce further misalignment error.

### 4.4. Future Opportunities

Current RGB-D SLAM algorithms are designed primarily for use within built environments and indoors, where the sensors components operate best. The development of SLAM algorithms specific to forest environments would likely assist in increasing the accuracy of object identification, and therefore the likelihood and frequency of loop closures, used to correct for positional drift. Such algorithms may potentially include a pre-processing noise filtration approach, akin to the iPad ARkit SLAM, to assist in depth frame registration, by simplifying the geometry present within the scene. Once registration is complete, the filtered information may then be re-projected back into the scene, based off camera geometry, to provide observations of finer vegetative structures. Further post-processing strategies, such as cylinder identification after stem segmentation, might then be used to further correct SLAM drift, by correcting overlapping observations of stem hemispheres that fall within a specific distance of one another and have similar stem diameters, identifying them as observations of the same stem. An alternative approach to reducing the effect of incidental duplicate stem captures and SLAM drift, may be the process of classifying individual points relative to the time they were observed within the capture process. Points may then be filtered from a stem observation if they are classified with a capture time outside a specific window of deliberate observation.

Such approaches, however, are reliant on access to raw data streams that are not locked to a proprietary SLAM algorithm. Whilst the iPad performed the best across both forest environments investigated within this study, with integrated RGB-D devices likely offering the best user experience when it comes to data capture and visualisation, there is limited access to depth images and inertial information. Future integrated devices may offer greater accessibility to RGB-D recordings, but until then, a potential workaround may be the utilisation of two RGB-D devices, one integrated and one standalone, located at a fixed distance, operating on the same RGB-D sensor technology, and with similar components (e.g., iPad and Kinect). This means that, where one device registers depth images in near real time and provides capture information to the operator, the other device records raw RGB-D data for processing at a later date, using an algorithm and approach of choice. This may also allow for further diagnosis of error, propagating within both depth images and registration workflows.

Currently, the relatively short range of RGB-D sensors integrated into handheld devices limits their inventory capture to measurements of basal stem structures and understory vegetation characteristics. Therefore, future research should investigate if the implementation of multiple low-cost technologies and platforms, such as consumer drones, can be used to reduce measurement error and/or increase the range of forest inventory metrics that can be acquired using cost-effective proximal RS approaches. Such an approach may also help to overcome the limitations associated with drone SfM approaches, that struggle to characterise vertical stem characteristics, due to occlusion resulting from upper canopy vegetation [44].

Since data capture for this study was conducted with the iPad device, there has been the release of the Reality Scan application [45]. This software uses RGB images from the device and a cloud based SfM workflow to generate point cloud representations of the device’s environment. As a part of this process, the application uses depth measurements from the RGB-D sensor and augmented reality feedback through the screen of the device, to inform the operator of image capture location and assist in ensuring adequate camera geometry and overlap between images, in near real time. However, at the time of publication, there are limitations on the number of RGB images that can be considered within a single project, potentially limiting the area of interest that can be captured with this approach. Regardless, how this application functions within a forested environment should be explored, even if only conducted at the individual stem scale, to potentially overcome some range and misalignment limitations associated with ToF RGB-D technologies.

Lastly, whilst inter-comparison between RGB-D devices is complex, due to optimal operating procedures varying due to RGB-D sensor technology and error propagating both within raw depth frames and the SLAM co-alignment algorithm, there should be the design of a method to benchmark current and emerging low-cost devices. This would assist in the operational adoption of RGB-D devices, as it may be used to inform their selection and use within different forest environments.

## 5. Conclusions

The results of this paper, suggest that the selection of current RGB-D devices assessed are more capable of operating within structurally complex forest environments, when compared to their predecessors. Whilst misalignment error due to incidental captures was visible in point cloud representations of stems, it was not observed to have a significant effect on DBH measurements derived with these devices. Individual captures of stems, as opposed to a continuous plot-scale capture, resulted in lower DBH RMSE. Furthermore, the complexity of the native woodland plots also resulted in complete loss of spatial positional estimates within the SLAM algorithms associated with the Kinect and Zed devices. This resulted in point cloud representations of forest structure no longer being reconstructed beyond this point. However, beyond one occurrence of self-occlusion with the Kinect sensor, it is difficult to determine the exact cause for this.

Of the sensors assessed, the iPad achieved the lowest DBH RMSE across both parkland and native woodland environments, and using both capture approaches. It also provided the best user experience, being integrated into a tablet, providing near real-time feedback of registration. Future research should focus on the development of bespoke SLAM algorithms for forest environments, methods for integrating multiple low-cost remote sensing solutions to increase the accuracy and range of forest representation, and the continued exploration of emerging consumer remote sensing technologies and approaches.

## Figures and Tables

**Table 1:** Stem counts, Please confirm color on table and please confirm table format. diameter at breast height (DBH) characteristics, and dominant species within site one (S1), at Royal Park (urban parkland), and site two (S2), at the You Yangs National Park (native woodland).

Site	Stem Count	DBH Mean	DBH IQR	Dominant Species
S1—Royal Park	60	34.9 cm	47.8 cm	Ironbark (Eucalyptus *sideroxylon*) River Red Gum (Eucalyptus *camaldulensis*) Yellow Gum (Eucalyptus *leucoxylon*)
S2—You Yangs	79	23.1 cm	19 cm	Yellow Gum (Eucalyptus *leucoxylon*)

**Table 2:** The number of stems successfully represented, and the root-mean-square error (RMSE) and bias of the stem diameter at breast height (DBH), for the colour and depth (RGB-D) devices at both site 1 (S1) and site 2 (S2). The * symbol denotes that some stems were unable to be captured beyond a point within the plot, due to loss of positional estimate within the SLAM spatial mapping process.

RGB-D Sensor	Individual Capture			Continuous Capture		
	Stems Captured	DBH RMSE	DBH Bias	Stems Captured	DBH RMSE	DBH Bias
	Site 1: Urban Parkland					
Microsoft Azure Kinect	57/60	3.72 cm	3.1 cm	56/60	4.96 cm	2.5 cm
Apple iPad Pro 2020	60/60	2.17 cm	−0.9 cm	60/60	3.32 cm	−0.9 cm
Stereolabs Zed 2	54/60	2.82cm	−1.3 cm	47/60	3.98 cm	−1.4 cm
	Site 2: Native Woodland					
Microsoft Azure Kinect	74/79	3.34 cm	2.7 cm	48/79 *	3.91 cm	3 cm
Apple iPad Pro 2020	76/79	2.15 cm	−1.1 cm	70/79	3.13 cm	−2.2 cm
Stereolabs Zed 2	66/79	3.89 cm	0.1 cm	28/79 *	3.62 cm	−0.9 cm

## Data Availability

Data presented in this study is openly available in FigShare at 10.6084/m9.figshare.c.6443699. Due to the large size of the associated point-cloud files some data has been omitted from this repository, but is available upon request.

## Abbreviations

The following abbreviations are used in this manuscript:

3D	Three-Dimensional
LiDAR	Light Detection and Ranging
TLS	Terrestrial Laser Scanning
PLS	Personal Laser Scanning
LC3D	Low-Cost Three-Dimensional
SfM	Structure from Motion
RGB-D	Colour and Depth
ToF	Time-of-Flight
IMU	Inertial Measurement Unit
SLAM	Simultaneous Localisation and Mapping
DBH	Diameter at Breast Height
RMSE	Root-Mean-Square Error
FoV	Field of View
AGH	Above Ground Height
S1	Site One
S2	Site Two
PS	Passive Stereo
NIR	Near Infrared
LSR	Least Squares Regression
RMSPE	Root-Mean-Square Percentage Error
PBH	Perimeter at Breast Height
